# Brain–body dysconnectivity: deficient autonomic regulation of cortical function in first-episode schizophrenia

**DOI:** 10.1017/S0033291724003428

**Published:** 2025-02-04

**Authors:** Kaia Sargent, Emily Martinez, Alexandra Reed, Anika Guha, Morgan Bartholomew, Caroline Diehl, Christine Chang, Sarah Salama, Kenneth Subotnik, Joseph Ventura, Keith Nuechterlein, Gregory Miller, Cindy Yee

**Affiliations:** 1 Department of Psychology, University of California, Los Angeles, Los Angeles, CA, USA; 2 Department of Psychiatry and Biobehavioral Sciences, University of California, Los Angeles, Los Angeles, CA, USA

**Keywords:** autonomic function, cognition, heart rate variability, neural oscillations, oscillatory coupling, schizophrenia

## Abstract

**Background:**

An accumulating body of evidence indicates that peripheral physiological rhythms help regulate and organize large-scale brain activity. Given that schizophrenia (SZ) is characterized by marked abnormalities in oscillatory cortical activity as well as changes in autonomic function, the present study aimed to identify mechanisms by which central and autonomic nervous system deficits may be related. We evaluated phase-amplitude coupling (PAC) as a physiological mechanism through which autonomic nervous system (ANS) and central nervous system (CNS) activity are integrated and that may be disrupted in SZ.

**Methods:**

PAC was measured between high-frequency heart rate variability (HF-HRV) as an index of parasympathetic activity and electroencephalography (EEG) oscillations in 36 individuals with first-episode SZ and 38 healthy comparison participants at rest.

**Results:**

HRV-EEG coupling was lower in SZ in the alpha and theta bands, and HRV-EEG coupling uniquely predicted group membership, whereas HRV and EEG power alone did not. HRV-EEG coupling in the alpha band correlated with measures of sustained attention in SZ. Granger causality analyses indicated a stronger heart-to-brain effect than brain-to-heart effect, consistent across groups.

**Conclusions:**

Lower HRV-EEG coupling provides evidence of deficient autonomic regulation of cortical activity in SZ, suggesting that patterns of dysconnectivity observed in brain networks extend to brain–body interactions. Deficient ANS–CNS integration in SZ may foster a breakdown in the spatiotemporal organization of cortical activity, which may contribute to core cognitive impairments in SZ such as dysregulated attention. These findings encourage pursuit of therapies targeting autonomic function for the treatment of SZ.

## Introduction

Schizophrenia (SZ) is characterized by widespread changes in the organization of brain networks, leading the disorder to be described as a syndrome of dysconnectivity (Friston & Frith, 1995). In a recent study involving healthy individuals, Raut et al. (2021) demonstrated that autonomic nervous system (ANS) activity plays a critical role in spatiotemporally organizing large-scale central nervous system (CNS) activity. Given prominent changes in ANS functioning in SZ, we hypothesized that autonomic dysfunction reflected in parasympathetic activity contributes to aberrant cortical activity and organization. The present study examined ANS modulation of CNS activity in SZ by measuring coupling between heart rate fluctuations and electroencephalography (EEG) oscillations. We sought to evaluate ANS–CNS integration to determine whether ‘dysconnectivity’, typically a description of impaired communication within or between brain networks, may also manifest in disrupted brain–body interactions.

Reports of autonomic changes in SZ date to Kraepelin, who described alterations in heart rate, skin conductance, and pupillary function as prominent characteristics of the disorder (Kraepelin, 1899; Stogios et al., 2021). These observed differences imply higher sympathetic activity and lower parasympathetic activity in SZ. More recent evidence indicates that autonomic disruption can lead to severe health complications, as individuals with SZ have a 15–20 year reduced life expectancy that is largely attributable to a threefold increase in risk for cardiovascular disease (Hennekens, Hennekens, Hollar, & Casey, 2005).

In addition to physical health consequences of autonomic dysfunction, there is speculation that ANS changes precipitate the development of psychotic symptoms. Studies of electrodermal activity indicate that autonomic changes temporally predict fluctuations in clinical symptoms associated with SZ (Dawson et al., 2010; Dawson & Nuechterlein, 1984; Dawson & Schell, 2002; Schell et al., 2005), suggesting that ANS changes contribute to symptom course. Autonomic dysfunction may also result in psychological changes that eventually lead to psychotic symptoms. For example, it has been suggested that chronically elevated arousal may be misinterpreted as fear signals and misattributed to (nonexistent) threats in the environment, leading to paranoid cognitions (Williams et al., 2004). Misattribution of visceral signals may also relate to interoceptive abnormalities and a disrupted sense of self (Yao & Thakkar, 2022), a core phenomenological feature of schizophrenia. Although psychological mechanisms linking autonomic dysfunction to psychotic symptoms have some empirical support and intuitive appeal, further work is needed to clarify these processes and the physiological mechanisms through which autonomic function relates to clinical symptoms in SZ.

In particular, the parasympathetic branch of the ANS has been linked to functioning across cognitive, affective, and social domains that are notably disrupted in SZ (Bär et al., 2005; Chung et al., 2013; Clamor, Lincoln, Thayer, & Koenig, 2016; Jáuregui et al., 2011). Parasympathetic function can be indexed reliably by measuring cyclical fluctuations in heart rate associated with respiration (0.15–0.4 Hz), known as high-frequency heart rate variability (HF-HRV). HF-HRV is widely theorized to index an individual’s ability to respond flexibly and adaptively to environmental demands (Porges, 2007; Thayer & Lane, 2000) and is consistently found to be lower in SZ (Clamor, Lincoln, Thayer, & Koenig, 2016). In clinical and non-clinical populations, higher HF-HRV is associated with better psychological function across various domains, including emotion regulation, ability to cope with stress, and overall mental well-being (Beauchaine & Thayer, 2015). Parasympathetic control is also thought to be highly relevant for social behaviors (Porges, 2001), and individuals with SZ have been observed to show decreased HF-HRV during social cognition tasks relative to resting baseline (Jáuregui et al., 2011).

Multiple studies of SZ have also found that low HF-HRV is associated with more severe positive and negative symptoms of psychosis (Bär et al., 2005; Chung et al., 2013; Kim, Ann, & Lee, 2011). Additionally, factor analytic approaches reveal a strong inverse relationship between HF-HRV and a cognitive/disorganized factor (Kim, Ann, & Lee, 2011). Associations between HF-HRV and cognitive symptoms in SZ are consistent with a large body of evidence obtained in non-clinical populations, supporting a relationship between higher HF-HRV and better cognitive performance, particularly involving executive function (Forte, Favieri, & Casagrande, 2019).

Neuroimaging studies of healthy individuals show HF-HRV to be associated with brain activity in prefrontal regions involved in attention, working memory, executive function, and social cognition (see Thayer, Hansen, Saus-Rose, & Johnsen, 2009). SZ is characterized by marked deficits in all of these domains as well as in prefrontal activity and connectivity (Perlstein, Carter, Noll, & Cohen, 2001; Smucny, Dienel, Lewis, & Carter, 2022). Additionally, evidence suggests that impairments in executive functioning and social cognition are associated with lower HF-HRV in SZ (Hamilton et al., 2014; Jáuregui et al., 2011; Kim et al., 2019; Mathewson, Jetha, Goldberg, & Schmidt, 2012).

Observing that the same brain structures and networks are involved in autonomic and behavioral regulation, Thayer and Lane (2000) proposed the neurovisceral integration model to describe a single integrated system of ANS and CNS feedback loops that accounts for robust relationships between HRV and psychological function. This system, termed the ‘central autonomic network’ (CAN), is comprised of prefrontal, cingulate, and subcortical regions that collectively support autonomic, cognitive, and emotional control. Within this framework, a few studies have investigated connections between ANS and CNS activity in SZ using measures of HRV (Schulz, Bolz, Bär, & Voss, 2016; Schulz, Haueisen, Bär, & Voss, 2019). These investigations relied on various linear (e.g. partial directed coherence) and nonlinear (e.g. transfer entropy) measures to investigate coupling between EEG and heart rate, respiration, and blood pressure time series. They observed altered CNS influence on autonomic function in SZ, though patterns were largely inconsistent even within studies; by some measures, SZ showed stronger EEG influence over HRV than did healthy comparison participants, and by other measures they showed weaker EEG to heart rate effects. These discrepancies may be due to the complex nature of ANS–CNS interactions; as the systems are comprised of multiple embedded feedback loops, some measures of coupling may reflect information transfer within specific circuits and timescales, while other measures reflect different (perhaps reciprocal) processes. Furthermore, the influence of factors such as attention may affect the direction of information transfer, leading to mixed results.

Whereas the above studies largely emphasized the role of top-down CNS control over peripheral physiology, recent work indicates that bottom-up ANS regulation of brain function may also contribute to the robust relationships between HRV and psychological function. Raut et al. (2021) demonstrated that autonomic fluctuations serve a fundamental role in organizing large-scale cortical activity. They showed that fMRI signal fluctuations in humans correspond to the phase of autonomic cycles indexed by respiratory volume, HRV, and pupil size. Moreover, these signal fluctuations map onto the structure of functional neural networks, suggesting that waves of sympathetic and parasympathetic activity help spatiotemporally structure brain activity into segregated networks by producing coherent activity in disparate brain regions that varies systematically as a function of autonomic cycle phase. Consistent with these findings, we recently demonstrated that HF-HRV oscillations can modulate EEG oscillations through phase-amplitude coupling (PAC; Sargent et al., 2024).

PAC is a fundamental mechanism of organization and communication typically studied in the brain whereby the phase of a slower oscillation (e.g. theta) modulates the amplitude of a faster oscillation (e.g. gamma), fostering the integration of neural assemblies across spatiotemporal scales. Although PAC has primarily been examined among neural oscillations, our findings add to a growing body of evidence that slower peripheral physiological cycles such as respiration and gastric rhythms also modulate the amplitude of EEG oscillations (Herrero et al., 2018; Richter, Babo-Rebelo, Schwartz, & Tallon-Baudry, 2017; Zelano et al., 2016). Unlike other measures of coupling in complex systems, PAC reflects a specific physiological mechanism such that slow oscillations affect faster oscillations by modulating the excitability of neural populations. Evidence that PAC extends to brain–body interactions suggests that autonomic activity plays a critical role in regulating cortical and psychological function.

Given evidence that autonomic cycles help regulate and organize CNS activity, we sought to determine whether dysfunction involving the parasympathetic system contributes to disorganized cortical activity in SZ. SZ is characterized by changes in oscillatory activity and synchrony across multiple EEG frequency bands (Uhlhaas & Singer, 2010). As a general principle, slower EEG rhythms cover larger areas of cortex and thus bring distant brain regions into communication, whereas high-frequency activity within these regions performs faster local computations and ‘binds’ stimulus features into a unified percept (Tallon-Baudry & Bertrand, 1999). Oscillatory abnormalities, particularly in the gamma frequency band, have been shown to correlate strongly with positive symptoms (Spencer et al., 2004). As coherent oscillatory activity is thought to enable information sharing and perceptual binding (Fries, 2015), aberrant oscillatory activity may disrupt communication between neural assemblies and contribute to a wide range of abnormalities in perception and thinking (e.g. hallucinations and delusions) due to a failure to effectively integrate information across brain regions and networks. Furthermore, individuals with SZ show global alterations in fMRI network connectivity (Pettersson-Yeo et al., 2011). These changes affect within- and between-network connectivity to such an extent that unifying pathophysiological mechanisms may be necessary to explain such a generalized breakdown in organization.

Building upon evidence that autonomic fluctuations help spatiotemporally segregate and organize functional brain networks in healthy individuals (Raut et al., 2021), the present study examined HRV-EEG coupling in SZ for evidence that ANS dysfunction contributes to aberrant CNS activity. We suggest that autonomic oscillations indexed by HF-HRV help regulate cortical excitability and coordinate oscillatory communication in the brain and that disrupted HF-HRV oscillations in SZ may lead to ineffective communication between neural assemblies and breakdowns in spatiotemporal organization. Specifically, we hypothesized that PAC between HF-HRV and EEG oscillations is lower in individuals with SZ than in healthy comparison (HC) participants and that Granger causality analyses would reveal decreased directional effects from cardiac to cortical activity. We further hypothesized that deficits in HRV-EEG coupling would be associated with symptom severity and cognitive dysfunction, particularly measures of executive function and social cognition, given known relationships between HRV and these domains (Jáuregui et al., 2011; Kim et al., 2019; Mathewson, Jetha, Goldberg, & Schmidt, 2012). Evidence of deficient autonomic regulation of neural activity in SZ would indicate that patterns of dysconnectivity extend to brain–body interactions and could partially account for global breakdowns in communication within and between brain networks.

## Methods and materials

### Participants

The study included 36 outpatients in the first-episode phase of schizophrenia (*n* = 35) or schizoaffective disorder (*n* = 1) and 38 healthy comparison participants (HC) recruited from the greater Los Angeles community. The HC sample was selected to be demographically comparable overall to the patient sample in age, gender, race/ethnicity, and parental educational level. SZ participants were being treated at the UCLA Aftercare Research Program and were recruited as part of a clinical trial of cognitive training and aerobic exercise (Nuechterlein et al., 2023). All physiological recordings for the present study were obtained after initial clinical stabilization of patients with antipsychotic medications and prior to randomization to a study intervention protocol.

Study inclusion criteria for the patients were: (1) an onset of psychotic illness within the last 2 years; (2) a DSM-5 diagnosis of schizophrenia, schizoaffective disorder (depressed type), or schizophreniform disorder; (3) 18 to 45 years of age; and (4) sufficient fluency in English to avoid invalidating research measures. Exclusion criteria for the patients were: (1) a known neurological disorder or significant CNS traumatic injury; (2) moderate or severe alcohol or substance use disorder within the 6 months prior to the first episode or evidence that substance abuse makes the schizophrenia diagnosis ambiguous; and (3) premorbid IQ below 70. The Structured Clinical Interview for *DSM-5*, Research Version (SCID-5-RV; First, Williams, Karg, & Spitzer, 2015) was used to determine diagnosis for SZ participants and eligibility for HC participants. HC participants were recruited through flyers distributed across Los Angeles area community sites and excluded if they had a history of any SZ spectrum or other psychotic disorders, current or recurrent major depression, bipolar disorder, obsessive-compulsive disorder, post-traumatic stress disorder, neurological disorders, significant head injury, or moderate to severe alcohol/substance use disorder history. Data from a subset of the HC group (*n* = 37) were analyzed in Sargent et al. (2024).

Participants provided written informed consent, and the UCLA Institutional Review Board approved all procedures. Participants were asked to refrain from drug or alcohol use within 24 hours of the laboratory visit and to refrain from smoking cigarettes or consuming caffeine at least 1 hour prior to the visit.

### Psychophysiological recording

Participants completed a 5-minute eyes-open resting state EEG subsequent to an auditory task (not analyzed in the present study). Participants were instructed to keep their eyes open and maintain their gaze on a white cross at the center of a black screen. EEG data were recorded at 2000 Hz using a Brain Products actiCHamp active electrode system with a 10–5 distribution of 90 scalp electrodes (including mastoids) and 6 EOG sites. Vertical and horizontal electrooculograms (EOGs) were recorded from electrodes placed above and below each pupil and adjacent to the outer canthus of each eye. EOG and EEG sites were referenced online to the left mastoid and re-referenced offline to average mastoids (Miller, Lutzenberger, & Elbert, 1991). Electrode impedances were kept below 25 kΩ per vendor guidance. EKG was recorded concurrently at 2000 Hz with electrodes placed symmetrically on the right and left lower ribs and a ground electrode located approximately two inches below and medial to the electrode on the side corresponding to the participant’s non-dominant hand.

### Symptom evaluation and cognitive testing

For all SZ participants, symptom severity was evaluated by trained clinicians using the Scale for the Assessment of Positive Symptoms (SAPS; Andreasen, 1984) and the Scale for the Assessment of Negative Symptoms (SANS; Andreasen, 1989). Functional outcome was assessed using the Role Functioning Scale, which contains four subscales measuring work/school productivity, independent living/self-care, relationships with family, and relationships with friends (Goodman, Sewell, Cooley, & Leavitt, 1993). A summary score was computed as the average of the four scores. SZ and HC participants completed the MATRICS Consensus Cognitive Battery (MCCB; Nuechterlein et al., 2008). The MCCB was designed specifically to assess cognitive domains that are impaired in SZ and includes measures of processing speed, attention/vigilance, working memory, verbal learning, visual learning, reasoning and problem solving, and social cognition. Given evidence of particularly strong relationships between HRV and social and executive functioning, analyses focused on the attention/vigilance, working memory, and social cognition domains of the MCCB.

### EKG processing

EKG data were initially processed using QRSTool (Allen, Chambers, & Towers, 2007). Automated beat detection was used to identify each R-wave, and trained research assistants visually inspected and corrected each timeseries for missed beats. The corrected interbeat interval (IBI) timeseries was transformed from cardiac time to real time to provide temporal alignment between the IBI and EEG timeseries. This was performed by calculating a vector of IBI values, one per millisecond, with each entry being the duration of the IBI during which that time point occurred. Each IBI timeseries was then subjected to autoregressive modeling using Kubios software (Niskanen, Tarvainen, Ranta-Aho, & Karjalainen, 2004) to identify the peak in the power spectrum in the HF-HRV range (0.15–0.4 Hz). Each participant’s HF-HRV peak-power frequency was used to extract sinusoidal variation in the participant’s heart rate (HF-HRV) at that frequency.

### EEG processing

Trained research assistants visually inspected EEG data for myogenic artifact and other noise. Artifactual segments and noisy channels were removed from the data, and a 1 Hz high-pass filter was applied, after which independent component analysis (ICA) was conducted using the Extended Infomax algorithm implemented in EEGLAB (DeLorme & Makeig, 2004) with the principal component decomposition limited to the first 30 components. All components were visually inspected by the first author. Artifactual components corresponding to eye blink, eye movement, and cardiac activity were identified and removed. The resulting ICA weights excluding artifactual components were then applied to the original dataset (without artifactual segments removed and without the 1 Hz high-pass filter) to preserve the temporal structure of the data. The data were reconstructed and down-sampled to 1000 Hz.

### Phase-amplitude coupling

Each participant’s real-time IBI timeseries was filtered around their HF-HRV peak frequency (±0.05 Hz), foregrounding the HF-HRV oscillation. A finite impulse response (FIR) filter was designed (Delorme & Makeig, 2004) with its order set to capture 3 cycles of the lower bound frequency for each participant. The filter was applied with a Hamming window point-by-point over the length of the timeseries. The filtered timeseries was then Hilbert-transformed to compute a timeseries of phase angles for the HF-HRV oscillation. The EEG timeseries was averaged over select frontal channels (Fz, FCz, F1, F2, and AFz) into a single timeseries, which was then filtered for each frequency band (delta: 1–4 Hz; theta: 4–8 Hz; alpha: 8–12 Hz; beta: 12–30 Hz; gamma: 30–50 Hz). A FIR filter with a Hamming window was applied for each frequency band, with the filter order set to capture 3 cycles of the lower bound frequency for each band. Filtered timeseries were then Hilbert-transformed to obtain the amplitude envelope for each EEG frequency band.

For each participant and each EEG band, the HF-HRV phase timeseries and the EEG amplitude timeseries were used to compute a modulation index (MI) based on methods developed by Tort, Komorowski, Eichenbaum, and Kopell (2010). HF-HRV phase angles were sorted into 18 bins spanning -pi to pi, and the average EEG amplitude in each frequency band was calculated for each HF-HRV phase bin. If there is no PAC between frequencies, then there will be a uniform distribution of average EEG amplitude across HF-HRV phase bins. Deviations from a uniform distribution were quantified with the MI, with higher values (ranging between 0 and 1) indicating systematic variance in EEG amplitude over HF-HRV phase bins and, therefore, reflecting PAC. A Group × EEG band MANOVA evaluated whether SZ and HC differed in PAC.

Granger Causality analyses were performed to assess the direction of PAC relationships using methods similar to those described by Munia and Aviyente (2021). Granger Causality assesses the extent to which a timeseries *X* provides information that is useful in predicting future values of timeseries *Y.* Variable *X* is said to Granger-cause variable *Y* if past values of *X* and *Y together* provide better predictions of future *Y* values than do past values of *Y* alone. The same HF-HRV phase and EEG amplitude timeseries for each frequency band were used for Granger analyses in each direction (i.e. with HF-HRV phase and EEG amplitude as *X* and *Y* respectively, and then a second time with *X* and *Y* reversed). This resulted in a heart-to-brain *F*-statistic and a brain-to-heart *F*-statistic for each participant and each band. To assess whether SZ and HC groups differed in the directionality of effects, Granger *F*-statistics were compared with an ANOVA for each EEG frequency band with group as the between-subjects factor and direction (heart-to-brain vs. brain-to-heart) as the within-subjects factor.

Data from one SZ participant was excluded from analyses due to the use of beta blockers. All analyses were repeated controlling statistically for antipsychotic (AP) medications (chlorpromazine equivalents), which did not alter the results.

## Results

Demographic and clinical characteristics of study participants are presented in Table 1. The HC sample was well matched to the SZ group and differed only in years of education and MCCB scores.

**Table 1.** Demographic and clinical characteristics

	Schizophrenia patients (*n* = 36)	Healthy comparison participants (*n* = 38)
Age (years), M (SD)	22.4 (4.9)	21.1 (2.7)
Sex (male/female)	25/11	27/11
Education (years), M (SD)	13.5 (2.1)*	14.4 (1.43)*
Parental education (years), M (SD)	14.9 (3.7)	14.8 (4.2)
Race/Ethnicity, *N*		
Asian	4	6
Black	5	2
Hispanic	13	12
Other	3	7
White	11	11
Symptom measures		
SAPS total, M (SD)	9.3 (3.6)	–
SANS total, M (SD)	12.6 (3.7)	–
Cognitive functioning		
MCCB overall composite, M (SD)	32.4 (13.3)**	49.5 (10.5)**
Attention/Vigilance, M (SD)	38.3 (11.9)**	45.4 (9.5)**
Working memory, M (SD)	40.4 (13.1)**	52.2 (9.4)**
Social cognition, M (SD)	41.6 (13.1)**	57.9 (12.0)**
Role functioning		
RFS summary score	3.5 (1.1)	–
Work/school productivity	2.6 (1.6)	–
Independent living/self-care	3.3 (1.1)	–
Relationships with family	4.8 (1.3)	–
Relationships with friends	3.2 (1.7)	–
Medications, *N*		
Aripiprazole (Abilify)	8	–
Cariprazine (Vraylar)	1	–
Chlorpromazine (Thorazine)	1	–
Lurasidone (Latuda)	2	–
Olanzapine (Zyprexa)	9	–
Paliperidone (Invega)	5	–
Quetiapine (Seroquel)	3	–
Risperidone (Risperdal)	7	–
Chlorpromazine equivalent, M (SD)	330.1 (194.2)	–

*Note:* MCCB, MATRICS consensus cognitive battery; RFS, role functioning Scale; SANS, scales for the assessment of negative symptoms; SAPS, scale for the assessment of positive symptoms.

* *p* < .05;

** *p* < 0.1.

### Phase-amplitude coupling

**Figure 1.**
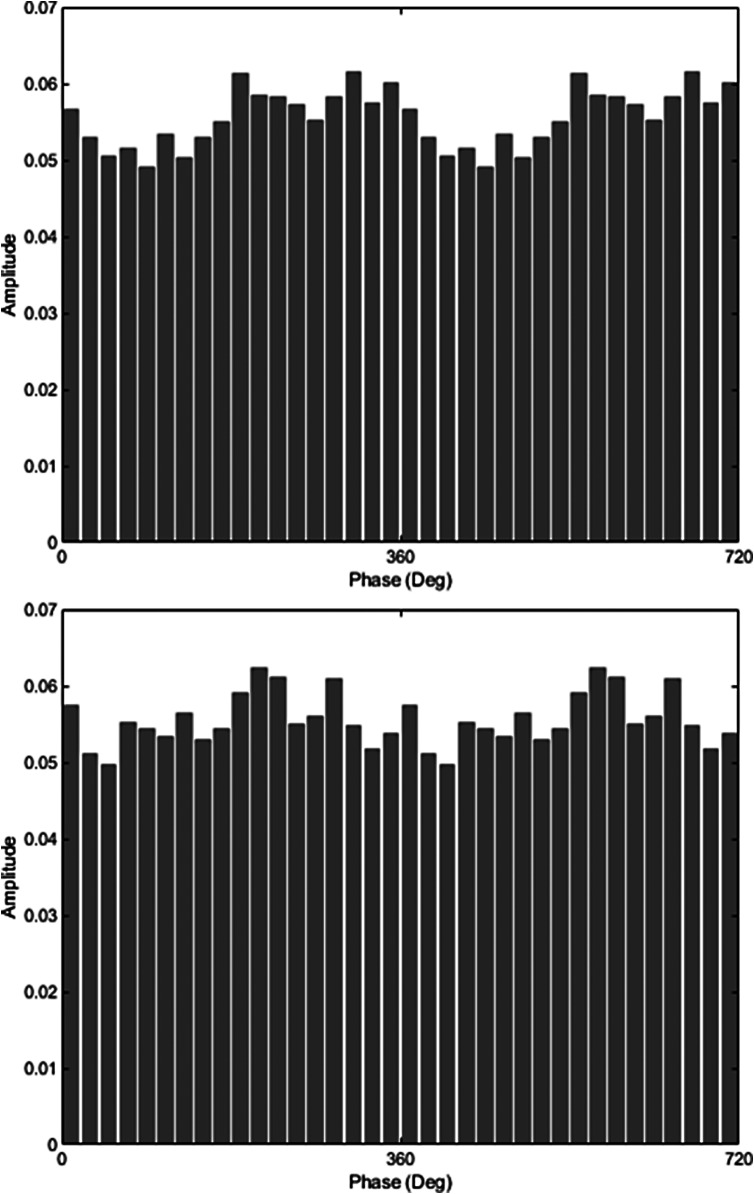
Average alpha amplitude in each HRV phase bin for (a) a representative HC participant (modulation index = 0.76e-3) and (b) SZ participant (modulation index = 0.50e-3).

**Figure 2.**
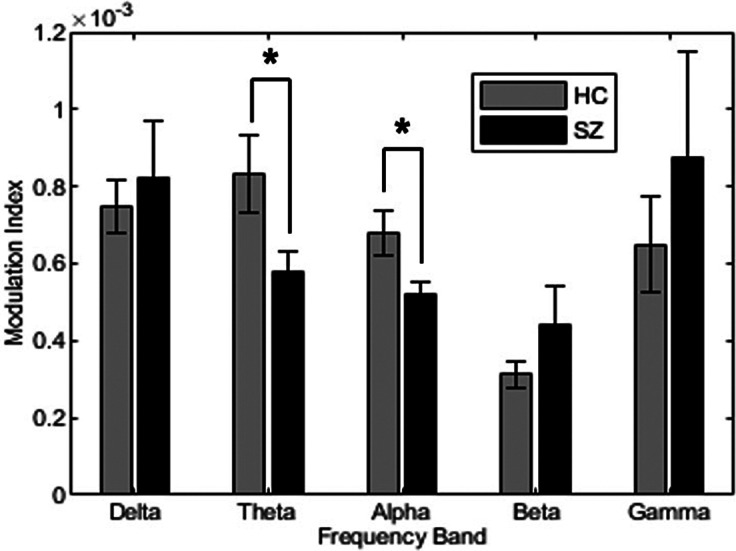
Average modulation index (MI) for each EEG frequency band for SZ and HC. Error bars reflect standard deviation. Theta MI and alpha MI were significantly lower in SZ as indicated by asterisks.

Figure 1 shows HF-HRV phase modulation of the EEG alpha rhythm in a representative HC participant (a) and SZ participant (b), who were selected as their MI values were close to the group average for HC and SZ, respectively. Sinusoidal fluctuations in alpha amplitude are visible in the HC plot and, though still apparent, are somewhat less orderly in the SZ plot. Figure 2 shows the average MI for each EEG frequency band in SZ and HC groups, with theta MI and alpha MI significantly lower in SZ.

A Group × EEG band MANOVA, evaluating whether SZ and HC differed in PAC, detected an effect of band (*F*(4,69) = 21.87, *p* = <.001, partial *η*^2^ = .56) with no main effect of group (*F*(1,72) < .01, *p* = .99, partial *η*^2^ < .001). Following up a Group × Band interaction (*F*(4, 69) = 2.62, *p* = .04, partial *η*^2^ = .13), simple effects tests revealed group effects for theta (*F* = 4.95, *p* = .03) and alpha (*F*(1,72) = 7.08, *p* = .01) but not for other frequency bands (delta: *F* = .23, *p* = .64; beta: *F* = 1.46, *p* = .23; gamma: *F* = .56, *p* = .46). In both theta and alpha bands, MI was higher in HC than SZ.

### Prediction of group membership

To determine whether HRV-EEG coupling contributed variance in diagnosis (HC vs. SZ) beyond that contributed by HF-HRV and EEG power, a binary logistic regression was performed predicting group membership from HF-HRV power, EEG power, and MI for each band that showed a group difference in HRV-EEG coupling (theta and alpha). Models were run separately for each band due to high collinearity between theta power and alpha power and between theta MI and alpha MI. The full models predicted considerable variance in diagnostic group (theta: chi-square (3) = 8.67, *p* = .03, Nagelkerke R^2^ = .15; alpha: chi-square (3) = 10.49, *p* = .01, Nagelkerke R^2^ = .18). For theta, MI contributed unique variance (∆R^2^ = .09, *p* = .04), whereas theta power and HF-HRV power did not (theta power: ∆R^2^ = .02, *p* = .29; HF-HRV: ∆R^2^ = .02, *p* = .25). For alpha, MI contributed unique variance (∆R^2^ = .11, *p* = .02), whereas alpha power and HF-HRV power did not (alpha power: ∆R^2^ = .02, *p* = .26; HF-HRV: ∆R^2^ = .04, *p* = .13).

### Relationships with clinical and cognitive functioning

Table 2 shows correlations between alpha and theta MI and scores on clinical measures, cognitive tests, and the Role Functioning Scale for SZ. Although MI was not associated with any of the clinical or role functioning measures, alpha MI and sustained attention were positively correlated. (Bonferroni correction would be overly conservative, as the four cognitive functioning correlations were not independent but would still provide a critical alpha (.05/4 = .0125) above the alpha/attention correlation *p*-value). When including CPZ equivalents as a covariate, the correlation between alpha MI and sustained attention remained significant (*r* = .39, *p* = .02) and no other correlations between symptom severity or cognitive functions changed substantially.

**Table 2.** Correlations between theta and alpha modulation index and cognitive, symptom, and functioning scores

	Theta MI		Alpha MI	
	*r*	*p*	*r*	*p*
SAPS summary score	.13	.53	−.19	.35
SANS summary score	.20	.33	−.22	.28
MCCB overall composite	.12	.50	.10	.57
Attention/vigilance	.09	.56	**.40**	**.01**
Working memory	.14	.41	.09	.61
Social cognition	−.04	.80	−.10	.56
RFS summary score	−.22	.22	.27	.13
Work/school productivity	−.33	.07	.25	.17
Independent living/self-care	−.14	.44	.07	.72
Relationships with family	−.24	.19	.09	.64
Relationships with friends	.03	.89	.34	.06

*Note:* MCCB, MATRICS consensus cognitive Battery; RFS, role functioning scale; SANS, scales for the assessment of negative symptoms; SAPS, scale for the assessment of positive symptoms.

### Granger causality

To assess directional relationships between HF-HRV and EEG oscillations and to determine whether SZ and HC groups differed in those relationships, Granger causality analyses were performed for each EEG frequency band using the same phase and amplitude timeseries that were used to compute MI. Table 3 presents the Group × Direction ANOVAs, which showed a stronger heart-to-brain effect than brain-to-heart effect in every band (as evidenced by estimated marginal means, not shown). There was no effect of Group and no Group × Direction interaction in any frequency band, indicating that both groups showed a predominant heart-to-brain effect.

**Table 3.** Results of ANOVAs evaluating effects of group (HC vs. SZ), direction (heart-to brain vs. brain-to-heart), and Group × Direction interactions on Gr

Frequency Band	Group		Direction		Group × Direction	
	*F*(1,72)	*p*-value	*F*(1,72)	*p*-value	*F*(1,72)	*p*-value
Delta	.32	.58	26.68	<.001	.27	.60
Theta	.95	.33	33.72	<.001	.87	.35
Alpha	.16	.69	24.92	<.001	.19	.67
Beta	.37	.54	13.63	<.001	.35	.56
Gamma	.08	.77	6.65	.01	1.04	.33

## Discussion

ANS and CNS dysfunctions have been reported extensively – though largely separately – in SZ. Present findings indicate that disrupted *integration* of ANS and CNS function may be an important physiological feature of SZ. Specifically, individuals with SZ showed less coupling between cardiac rhythms, as measured by HF-HRV, and neural oscillations in theta and alpha bands than HC participants. Furthermore, lower coupling in the alpha band correlated with the extent of deficit in attention/vigilance. In binary regression models, only lower HRV-EEG coupling, not HRV or EEG power, contributed unique variance as a predictor of group status. Although groups differed in the magnitude of coupling, the directions of effects were consistent, with both HC and SZ showing a predominant heart-to-brain effect.

Accordingly, dysconnectivity in SZ appears to extend beyond the brain to encompass peripheral physiology and aligns with recent evidence that autonomic cycles play a critical role in regulating and organizing CNS activity (Raut et al., 2021). We propose that deficient ANS–CNS integration in SZ fosters a breakdown in the spatiotemporal organization of cortical activity, which may contribute to core impairment in sustained attention in SZ.

### Mechanisms of ANS–CNS dysconnectivity in SZ

PAC supports the coordination of oscillatory activity within the brain. Slower oscillations (e.g. theta) modulate the excitability of neural assemblies that generate faster local rhythms (Canolty & Knight, 2010), fostering the integration of neural populations across spatiotemporal scales. Autonomic fluctuations may similarly help regulate cortical excitability and coordinate neural oscillations. Abnormal neural oscillations are widely believed to contribute to a central pathophysiological mechanism in SZ, as deficient oscillatory activity is closely correlated with cognitive dysfunction and other core symptoms of the disorder (Uhlhaas & Singer, 2010). A failure of autonomic regulation of cortical excitability may contribute to abnormalities in oscillatory activity and synchrony, resulting in compromised organization and communication in the brain that contributes to psychosis and cognitive dysfunction.

In addition to oscillatory abnormalities, SZ is characterized by altered patterns of connectivity in functional networks, which may also be related to autonomic dysregulation. Emerging evidence supports a role for autonomic activity in fostering connectivity within functional networks. Raut et al. (2021) demonstrated in nonpatients that traveling waves of autonomic activity produce coherent fMRI activity in disparate brain regions that varies systematically as a function of autonomic oscillatory phase and that patterns of activation map onto the structure of functional neural networks. We reported MI findings in the present HC sample (Sargent et al., 2024) that support the proposal by Mather and Thayer (Mather & Thayer, 2018) that heart rate oscillations induce or modulate oscillatory activity in the brain which, in turn, may enhance functional connectivity in networks that support cognition. Present data indicate less impact of heart rate oscillations on neural oscillatory activity in SZ. Further work is needed to determine whether HRV-EEG coupling tracks and perhaps drives functional connectivity and whether abnormal connectivity in SZ may be casually related to autonomic dysregulation.

### Autonomic and attentional regulation

The present finding that HRV-EEG coupling in the alpha band correlates with attention/vigilance is aligned with prominent models of ANS and CNS integration, such as the neurovisceral integration model (Thayer & Lane, 2000) and polyvagal theory (Porges, 1995). Both models emphasize relationships between autonomic function and attention, as regulation in both domains depends on shared anatomical structures and functional networks. Porges has reported extensively on reductions in HRV during sustained attention tasks (Porges, 1972, 1986; Porges & Raskin, 1969), with individuals with higher baseline HRV showing more HRV suppression to stimuli and better performance. Effective autonomic regulation may facilitate allocation of resources appropriate to environmental demands and conservation of resources when demands subside, leading to an improved ability to regulate and maintain attention.

Disrupted attention is a prominent feature of SZ that may have diverse consequences for stimulus processing and other cognitive functions (Braff, 1993; Nuechterlein & Dawson, 1984). Attentional dysfunction has been found to relate to deficient modulation of alpha rhythms in SZ (Kustermann et al., 2016), and although yet to be examined, it is possible that inefficient alpha modulation is related to disrupted autonomic regulation. As attentional deployment is critical for the selection of task-relevant inputs, attentional dysfunction in SZ may lead to disrupted stimulus filtering and other processing abnormalities across perceptual and cognitive domains (Kustermann et al., 2016).

### Limitations and future directions

Disruptions associated with the COVID-19 pandemic limited our ability to recruit a greater number of participants, which would have yielded large enough samples to examine within-group heterogeneity, especially among SZ. Given that all patients were receiving psychiatric treatment for an initial episode of illness from the same outpatient clinic, duration of illness was relatively constrained (median duration = 7 months) along with medication status and level of clinical care. Future work would benefit from recruitment of larger samples that would allow for exploration of any effects associated with different antipsychotic medications. Patients who participated in the present study were also relatively low in rated symptom levels, which might explain why HRV-EEG PAC was not related to positive or negative symptoms.

Due to the fact that SZ was the only diagnosis assessed, it remains unclear whether deficient ANS–CNS integration is specific to SZ or whether it may be a transdiagnostic phenomenon. Given that HRV changes are prevalent across the diagnostic spectrum, it is possible that lower HRV-EEG coupling contributes to other psychiatric disorders. As attentional dysfunction is also a common transdiagnostic feature across psychiatric diagnoses, it is possible that ANS–CNS dysconnectivity is more closely related to cognitive dysfunction than it is to specific psychiatric symptoms. Additional research is also needed to examine relationships between HRV-EEG coupling and cognitive, perceptual, and affective processes in diverse clinical and non-clinical populations to further clarify these relationships. Furthermore, as attention may fluctuate over the course of a session, it would be beneficial to assess state-level attentional effects in addition to trait-level effects as they relate to HRV-EEG coupling. Despite the noted limitations, findings from the present study provide a deeper understanding of ANS–CNS integration and its relevance for mental health and cognitive function which, in turn, may support and encourage pursuit of therapies targeting autonomic function in the treatment of SZ and potentially other disorders.
